# Coagulation of Hydrophobic Ionic Associates of Cetyltrimethylammonium Bromide and Carrageenan

**DOI:** 10.3390/molecules28227584

**Published:** 2023-11-14

**Authors:** Alexander Shyichuk, Dorota Ziółkowska, Joanna Szulc

**Affiliations:** Faculty of Chemical Technology and Engineering, Bydgoszcz University of Science and Technology, Seminaryjna 3, 85-326 Bydgoszcz, Poland; szyjczuk@pbs.edu.pl (A.S.); joakan@pbs.edu.pl (J.S.)

**Keywords:** CTAB, carrageenan, ionic associates, flocculation, particle size distribution

## Abstract

In aqueous solutions, cetyltrimethylammonium cations bind to carrageenan polyanions, and the resulting ionic associates form macroscopic aggregates due to hydrophobic interaction. At certain ratios of cetyltrimethylammonium to carrageenan, the resulting colloidal particles auto-flocculate. According to visual observations, the ratio ranges from 1 to 3 mmol/g; otherwise the suspensions are stable. By measuring the sedimentation rate and particle size distribution, the most extensive flocculation was found to be from 1.7 to 2.3 mmol/g. The ratio corresponding to the fastest auto-flocculation was precisely determined by titrating the reagents with small increments and recording the turbidity. The turbidimetric titration plots contain distinct break points corresponding to the most extensive flocculation. These break points occur at the same ratio of carrageenan to cetyltrimethylammonium over a wide range of reagent concentrations. The precise values of the critical ratio were found to be 1.78 and 1.53 mmol/g, respectively, during the titration of cetyltrimethylammonium with carrageenan and vice versa. The number of anionic sulfate groups in carrageenan was measured by ICP OES and found to be 1.35 mmol/g. This value is consistent with the critical ratio of the auto-flocculation.

## 1. Introduction

Cetyltrimethylammonium bromide (CTAB) is a cationic surfactant with numerous applications. Thanks to its antiseptic effect against bacteria and fungi, CTAB is used as a preservative in cosmetics; due to its positive charge, it acts as a smoothing component in hair conditioners [1]. In biology, it appears to be helpful in isolating DNA from inside the cell as well as for analysing proteins [2,3]. CTAB-based mixed micelles are useful in the delivery of antihistamine drugs [4]. Moreover, it is employed as a reagent for obtaining gold nanoparticles, which are used in medicine and other areas, e.g., optics, electronics or catalysis [5]. CTAB also helps in the synthesis of mesoporous adsorbents with a highly ordered structure [6]. Due to its toxicity, i.e., the burning effect on mucous membranes, as well as embryotoxic and teratogenic effects, the dosage of CTAB in products applied to the skin is limited to 0.1% [7]. CTAB’s reactivity is largely the result of the positive charge of its ions in solution. Cetyltrimethylammonium cation forms micellar ionic associates with anionic surfactants and anionic dyes [4,8]. Ionic associates of CTAB with hyaluronic acid anions form nanogels [9].

Carrageenan is a sulfated polysaccharide of marine macroalgae that has a wide range of applications. Carrageenan is used as a thickener, emulsion stabiliser and gelling agent in the food industry [10,11,12,13,14,15,16,17]; as a drug delivery agent and bone substitute and in wound dressings in biomedicine [18,19,20]; and as an ingredient in cosmetic products [21]. Depending on the seaweed source, carrageenans differ in the ratio of galactose and anhydro-galactose units and in the content of sulfate groups [22,23,24]. Due to multiple negative charges, carrageenan macromolecules interact strongly with inorganic and organic cations. The texture and properties of carrageenan gels depend on the Na, K, Mg and Ca cations present [25,26,27,28,29]. Cationic dyes form ionic associates with carrageenan polyanions, resulting in hydrophobic aggregation and spectral changes [30]. Polycations of poly-lysine and poly(diallyl-dimethylammonium) form strong ionic associates with carrageenan polyanions [31,32].

Phase separation studies as a function of mixing ratio are important for understanding the stability of both binary polyelectrolyte-surfactant micelles and ternary polyelectrolyte-surfactant-solid surface systems. For example, aggregates of oppositely charged polyelectrolytes and surfactants play an important role in hair care, oil extraction, food formulation, drug delivery, etc. [33]. Ionic associates of CTAB with polyacrylic acid effectively stabilise nanoclay dispersions [34].

Ion associates of cationic surfactants and carrageenan are hydrophobic and prone to coagulate or adsorb on solids [35,36]. The coagulation efficiency depends on the relative amount of organic cations and increases when the aliphatic tails are longer [36]. In this work, the coagulation behaviour of ionic associates of CTAB and carrageenan was investigated as a function of the component ratio. The sedimentation measurements made it possible to determine the range of component ratios ensuring fast coagulation. Additionally, a titration approach was used to precisely determine the ratio of carrageenan to cetyltrimethylammonium, which ensures the fastest coagulation. The point of fastest flocculation was determined by titrating the components in small increments while continuously measuring the turbidity of the suspension.

## 2. Results and Discussion

### 2.1. Sedimentation of the Ionic Associates

**Figure 1:**
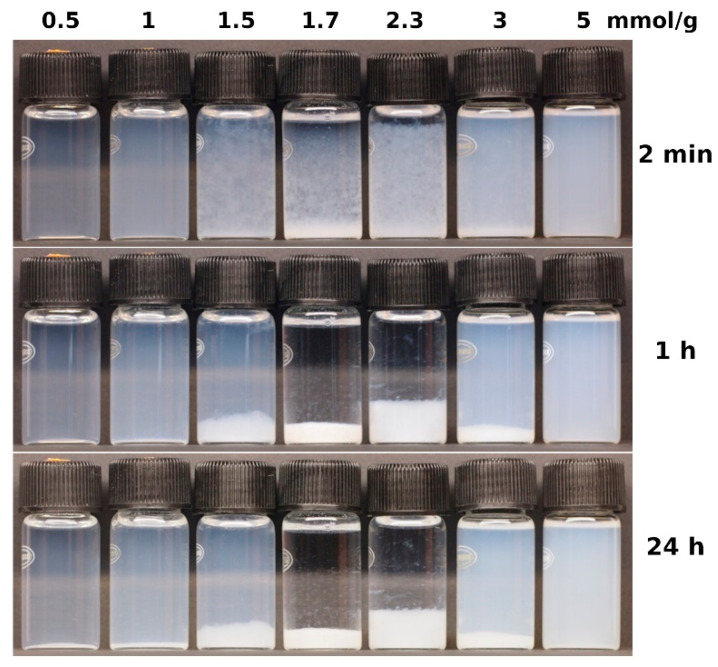
The colloidal stability of suspensions formed in aqueous solutions of CTAB and carrageenan. The ratio of CTAB to carrageenan and the sedimentation time are given.

When mixed, CTAB and carrageenan immediately formed turbid solutions (Figure 1, 2 min). The turbidity of the tested solutions indicates that phase separation is taking place. Hydrophobic ionic associates separate from the aqueous phase and scatter light (Figure 1, 2 min). Some of the resulting suspensions were stable, whereas others were not. The stable suspensions had a CTAB to carrageenan ratio of 1 mmol/g and less or 5 mmol/g and more (Figure 1, 1 and 24 h). At a CTAB to carrageenan ratio ranging from 1.5 to 3 mmol/g, visible flocs formed and slowly sedimented (Figure 1, 1 and 24 h). A very fast flocculation and sedimentation occurred at the ratio of CTAB to carrageenan of 1.7 mmol/g (Figure 1).

**Figure 2:**
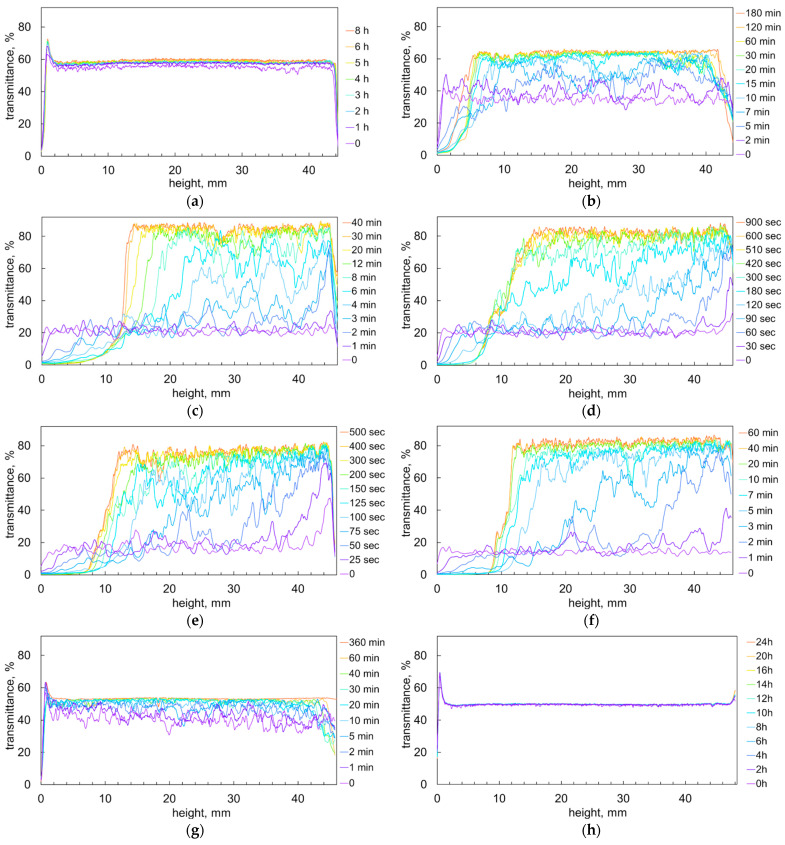
Profiles of light transmission in suspensions of CTAB–carrageenan ionic associates. CTAB to carragenaan ratio equals to (in mmol/g): (**a**) 1, (**b**) 1.5, (**c**) 1.7, (**d**) 1.8. (**e**) 2, (**f**) 2.3, (**g**) 3 and (**h**) 5.

A detailed study of sedimentation dynamics was carried out using a Turbiscan scanning turbidimeter. Figure 2 shows light transmission along the cuvette. The turbidity profiles recorded with time intervals reveal details of phase separation and particle sedimentation [32,37,38,39].

Figure 2a shows the Turbiscan graphs recorded of a suspension with a CTAB to carrageenan ratio of 1 mmol/g. The flat profiles of light transmittance have only slight fluctuations. It is obvious that the suspension remained stable over time. Over 8 h of monitoring, the light transmittance values show only a slight upward trend (Figure 2a). The CTAB ions likely rearranged along carrageenan molecules and formed more compact hydrophobic clusters.

Figure 2b shows the light transmittance profiles of a suspension with a CTAB to carrageenan ratio of 1.5 mmol/g. The large signal fluctuations suggest that large aggregates were present in the suspension. The horizontal scale shows that the fluctuations are 0.3 to 1 mm wide (Figure 2b). Presumably, this is the approximate size of the formed particles. The light transmittance increased over time, indicating that the number of suspended particles decreased. In the centre of the cell, the transmittance values increased from approximately 35% to 65% (Figure 2b). This suggests that about half of the particles have settled. A strong decrease in light transmission at the bottom of the cell indicates the formation of a precipitate [40]. The sediment layer was about 4–5 mm in height and became more compact over time (Figure 2b).

Figure 2c–f illustrate sedimentation in suspensions with ratios of CTAB to carrageenan of 1.7 to 2.3 mmol/g. The plots indicate clearly that the suspensions were highly unstable. The recorded transmittance profiles have large fluctuations, indicating that large flocs formed. Some of the fluctuations are several millimeters wide, suggesting that the flocs were a few millimeters in size. The rapid increase in transmittance values at the top of the cell indicates fast sedimentation of the flocs (Figure 2e,f). The fourfold increase in transmittance values suggests that approximately three fourths of the particles settled. The transmittance values at the bottom decreased, indicating the formation of precipitates (Figure 2c–f). The fastest sedimentation took only 9 min (Figure 2e).

Figure 2g shows the graphs corresponding to a suspension with a ratio of CTAB to carrageenan of 3 mmol/g. The graphs indicate rather small fluctuations and moderate changes in transmittance over time. The suspension with a CTAB to carrageenan ratio of 5 mmol/g was fully stable, and the corresponding transmittance graphs did not change for 24 h (Figure 2h).

**Figure 3:**
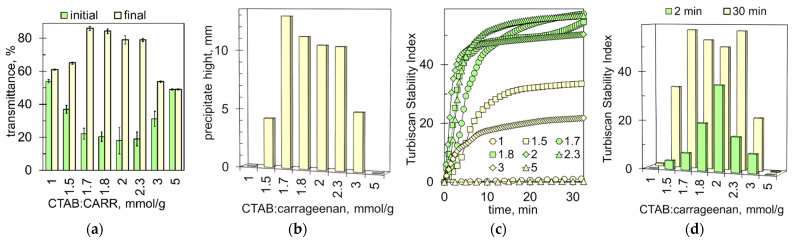
(**a**) Initial and final light transmittance values measured at the centre of the measuring cell and (**b**) sediment height as a function of CTAB to carrageenan ratio. (**c**) Changes in Turbiscan Stability Index over time at indicated CTAB to carrageenan ratios (in mmol/g). (**d**) The values of Turbiscan Stability Index after 2 and 30 min of sedimentation.

Figure 3a shows the initial and final values of light transmittance at the middle of the cell depending on the ratio of CTAB to carrageenan (CARR). These trends are clearly opposite. The lowest initial transmittance values and the highest final transmittance values were at CTAB to carrageenan ratios of 1.7 to 2.3 mmol/g (Figure 3a). In other words, these ratios resulted in the most turbid suspensions, which settled quickly. The trend in the amount of precipitate (Figure 3b) was similar to that in the transparency of the supernatant (Figure 3a). The more particles settled, the higher the sediment layer and the higher the final light transmittance. Figure 3c shows the Turbiscan Stability Index (TSI) over time. When the ratio of CTAB to carrageenan was beyond the range of 1 to 5 mmol/g, the TSI values increased slowly and did not exceed 3. Such low TSI values indicate that the tested suspensions are quite stable [39,41]. However, the TSI values rapidly increased over the first 10 min when the ratio of CTAB to carrageenan was in the range of 1.5 to 3. The maximum TSI values were between 20 and 60 (Figure 3c). Figure 3d shows the TSI values at the initial (2 min) and later (30 min) stages of sedimentation. The maximum TSI value at the initial phase of sedimentation (2 min) was for a ratio of CTAB to carrageenan of 2, while the maximum TSI values at the later stage (30 min) were for a CTAB to carrageenan ratio ranging between 1.7 and 2.3. It is likely that the relatively slow secondary aggregation of the particles resulted in quite similar TSI values at the final stage.

### 2.2. Shape and Size of Particles of Ionic Associates

**Figure 4:**
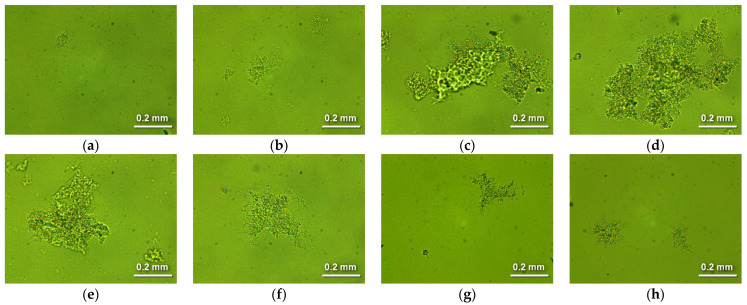
Micro-images of the suspended particles. CTAB to carragenaan ratio equals to (in mmol/g): (**a**) 1, (**b**) 1.5, (**c**) 1.7, (**d**) 1.8. (**e**) 2, (**f**) 2.3, (**g**) 3 and (**h**) 5.

Figure 4 shows micro-images of the particles/flocs in the studied suspensions. The changes in shapes and sizes of the particles confirm that auto-flocculation occurs. At a CTAB to carrageenan ratio of 1 mmol/g, the particles were small (30 to 100 μm) and compact (Figure 4a). At a CTAB to carrageenan ratio of 1.5 mmol/g, the particle sizes increased to 200 μm and larger (Figure 4b). At CTAB to carrageenan ratios of 1.7 to 2 mmol/g, very large flocs of 500 to 1000 μm were formed (Figure 4c–e). These proportions of reactants provided the fastest sedimentation (Figure 2 and Figure 3). The flocs were highly irregular in shape, indicating that they formed through the aggregation of many smaller particles (Figure 4c–e). At higher ratios of CTAB to carrageenan (2.3, 3, and 5 mmol/g), the particles were smaller, while the irregular shape remained (Figure 4f–h).

**Figure 5:**
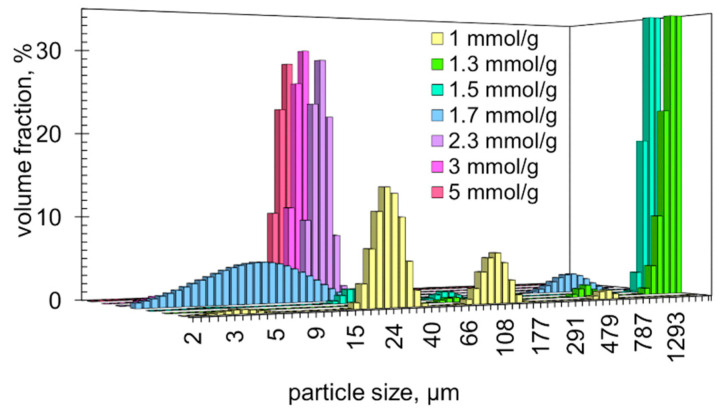
Particle size distributions of the suspensions with the indicated ratios of CTAB to carrageenan.

The size distributions of the suspended particles confirm these conclusions. At CTAB to carrageenan ratios of 1, 1.3, 1.5 and 1.7 mmol/g, the recorded particle size distributions were multi-modal (Figure 5). This fact confirms the occurrence of auto-flocculation. Multi-modal distributions are known to form when multi-level aggregation of particles occurs [32,42,43,44]. Suspensions with a CTAB to carrageenan ratio of 1.3 and 1.5 mmol/g contained large portions of very large particles (1570–1920 μm; 82% and 90%, respectively). This confirms the formation of flocs. However, the suspension with a CTAB to carrageenan ratio of 1.7 mmol/g mainly revealed particle sizes from 2 to 36 μm (Figure 5), which does not agree with the microscopic images (Figure 4c). This discrepancy can be explained by the fact that the particle size meter used does not register particles larger than 2000 μm. The particle size distributions of the suspensions with higher ratios of CTAB to carrageenan (of 2.3, 3 and 5 mmol/g) are unimodal and centred at 27 μm (Figure 5). This finding confirms that higher ratios of CTAB to carrageenan result in fairly stable suspensions.

### 2.3. Probable Mechanism of Auto-Flocculation

There are two basic phenomena underlying this auto-flocculation: (1) electrostatic interaction and (2) hydrophobic interaction. The electrostatic attraction is very strong due to the multiple charges of the ionic macromolecules [45,46]. The hydrophobic interaction is weaker and results in self-association of long alkyl tails of cetyltrimethylammonium. Similarly, combined electrostatic and hydrophobic interactions result in the formation of mixed micelles of anionic surfactants and cationic drugs [47].

**Figure 6:**
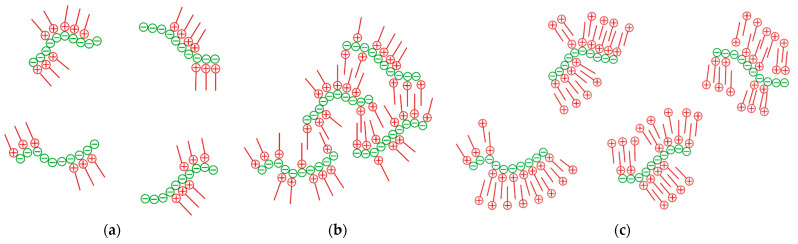
Scheme of ionic association at (**a**) insufficient, (**b**) equal and (**c**) excessive amounts of CTAB (red) to carrageenan (green).

At low ratios of CTAB to carrageenan, only part of the anionic sites on the carrageenan macromolecule are filled with cetyltrimethylammonium cations. The ionic associates are rather hydrophilic in nature due to the remaining negative charges. However, aggregates of oppositely charged polyelectrolytes and surfactants can slowly change their structure and properties [33]. The hydrophobic interaction pushes the cetyltrimethylammonium cations to rearrange along the anionic macromolecule to clump together their non-polar aliphatic tails (Figure 6a). As a result, compact micellar aggregates are formed, which are complexed with an anionic macromolecule [45]. These micellar aggregates are (sub)micrometre in size and therefore scatter light, causing the solution to become turbid (Figure 1).

When the amounts of cetyltrimethylammonium cations and carrageenan anionic units are approximately equal, the positive and negative ionic charges are neutralised. Being uncharged, the ionic associates do not interact with polar water molecules and lose solubility [45]. Due to the hydrophobic interaction of the alkyl chains, separate ionic associates attract each other and form large aggregates (Figure 6b). The resulting hydrophobic aggregates stick together again and again, causing macroscopic flocculation (Figure 1).

Figure 6c shows the case when the amount of cetyltrimethylammonium cations exceeds the amount of carrageenan anionic units. Excess cations are adsorbed on ionic associates providing a positive charge and thus polar interactions with water molecules. The resulting ionic associates form stable suspensions that strongly scatter light (Figure 1). The suspended particles have rather a compact shape (Figure 4g,h).

### 2.4. The Ratio of CTAB to Carrageenan That Provides the Most Intense Flocculation

In order to determine the conditions of the most intense flocculation, it is necessary to prepare and test multiple suspensions with varying ratios of CTAB to carrageenan. For this purpose, the titration technique is very convenient. Automatic titration in small steps along with continuous turbidity measurement allows for a precise determination of the onset of flocculation [32,48].

**Figure 7:**
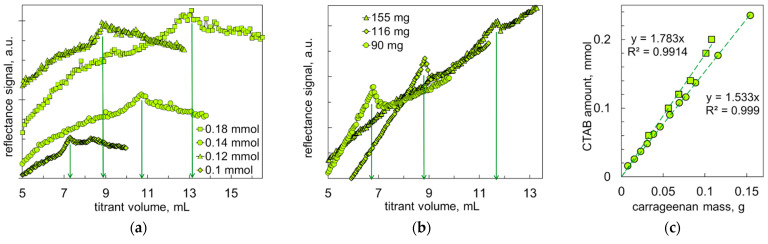
Turbidity changes under titration experiments: (**a**) the indicated amounts of CTAB were titrated with 8 g/L of carrageenan solution; (**b**) the indicated amounts of carrageenan were titrated with 20 mM of CTAB solution. (**c**) The amount of CTAB vs. the amount of carrageenan in the reaction mixture at the critical point. Quadrates correspond to the titration of CTAB with carrageenan and circles correspond to the titration of carrageenan with CTAB.

Figure 7a shows example turbidimetric graphs recorded during the titration of the CTAB solutions with the carrageenan solution. In the initial phase, the turbidity of the reaction mixture is constantly increasing, which is obviously caused by the increased number and size of the ionic associates being formed. When the amounts of CTAB and carrageenan charges are nearly equal, the particles start to flocculate. After this critical point, turbidity decreases because of the formation of large flocs (Figure 7a). In turn, Figure 7b shows the turbidimetric plots recorded during the titration of the carrageenan solutions with the CTAB solution. In general, the changes in turbidity are similar to those in Figure 7a. With equal number of CTAB and carrageenan charges, a distinct peak in turbidity was observed. Further addition of the CTAB solution caused the turbidity values to reach a local minimum and increase again.

Figure 7c shows the relationship between amounts of CTAB and carrageenan at the critical point of the titration graphs. The resulting graphs are strictly linear (R^2^ ≥ 0.999), indicating that the critical ratio is constant over a wide range of concentrations. The most intense flocculation occurred at a ratio of CTAB to carrageenan of 1.53 mmol/g during the titration of carrageenan with CTAB and of 1.78 mmol/g during the titration of CTAB with carrageenan. In other words, the titration experiments indicate that one gram of carrageenan contains 1.53 mmol or 1.78 mmol of sulfate groups. These values are in line with the sulphur content of 1.35 mmol/g, as measured by ICP OES [32].

### 2.5. Structure and Composition of Ionic Associates Investigated by XRD and FTIR Methods

**Figure 8:**
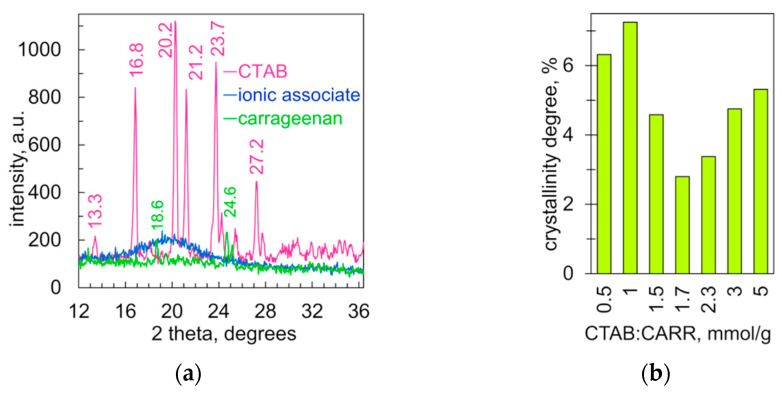
(**a**) X-ray diffractograms of CTAB, carrageenan and their ionic associate at a ratio of 1.5 mmol/g. (**b**) The degree of crystallinity of ionic associates vs. the ratio of CTAB to carrageenan (mmol/g).

The XRD data clearly show that there is a significant reduction in molecular order upon the binding of CTAB and carrageenan. The X-ray diffraction pattern of cetyltrimethylammonium bromide has a series of intense peaks (Figure 8a), indicating a well-ordered structure. The XRD pattern of carrageenan has three small peaks only (Figure 8a). Various X-ray diffraction patterns of carrageenan have been reported in the literature [49,50,51,52]. The carrageenan itself is likely to be almost amorphous, while the peaks are due to admixtures such as cellulose, sodium chloride, potassium chloride, etc. The low-ordered structure of carrageenan is probably due to its polymeric nature and high hygroscopicity. After binding CTAB and carrageenan, their diffraction peaks disappear completely. The diffraction pattern of the ionic associate contains only a wide halo in the 2θ range from 15 to 28 degrees (Figure 8a). Such a halo indicates that the tested material has a very low molecular order. Very similar XRD patterns were recorded for complexes of carrageenan and dodecyl-ammonium [53].

The degree of molecular order of the ionic associates was calculated as the ratio of the halo area to the background area in the X-ray diffraction pattern. The values range from 2.8 to 7.3% (Figure 8b). The minimum degree of crystallinity occurred when the ratio of CTAB to carrageenan was 1.7 mmol/g, which is close to equimolar (see Section 2.4).

**Figure 9:**
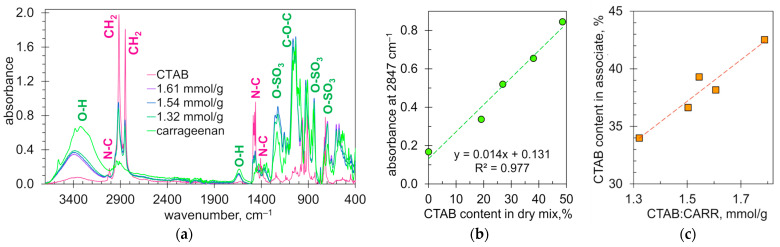
(**a**) ATR-FTIR spectra of CTAB, carrageenan and precipitated ionic associates at the indicated ratios of CTAB to carrageenan. (**b**) The absorbance of the characteristic peak at 2847 cm^−1^ vs. the CTAB content in dry mix of CTAB and CARR. (**c**) The content of CTAB (%) in the precipitated ionic associate vs. the ratio of CTAB to carrageenan in the reaction mixture (mmol/g).

Figure 9a shows the FTIR spectra of CTAB, carrageenan and their ionic associates. The CTAB spectrum contains peaks characteristic of alkyl chains and N–C bonds. Two strong sharp peaks at 2916 and 2849 cm^−^^1^ are due to the asymmetric and symmetric stretching of –CH_2_– groups [53,54]. The moderate peaks at 908 and 959 cm^−^^1^ are due to C–N stretching. The peaks at 1486, 1472, 1462 and 1406 cm^−^^1^ are also due to C–N bonds [54]. The peak at 3015 cm^−^^1^ may be due to the stretching of the trimethylammonium group [53].

The spectrum of carrageenan contains peaks characteristic of sulfated polysaccharides. The broad peak centred at 3400 cm^−^^1^ is well known to be characteristic of O–H bonds [53]. The peak at 1640 cm^−^^1^ corresponds to the OH bending. The peaks at 1220, 840 and 697 cm^−1^ correspond to the ester sulfate groups, O–SO_3_ [49,53]. The strong band with two peaks at 1036 and 1060 cm^−^^1^ is probably due to C–O–C bonds [49].

The spectra of the ionic associates contain peaks characteristic of both CTAB and carrageenan (Figure 9a). The peaks of hydroxyl groups (3400 and 1640 cm^−^^1^) and alkyl groups (2916 and 2849 cm^−^^1^) have reduced intensity. The obvious reason is the reduced content of the components compared to their pure forms. On the contrary, the peaks of ester sulfate groups at 1220 and 697 cm^−^^1^ increased in intensity. A possible cause is the ionic interaction between the ester sulfate groups and the CTAB cations. The ionic interaction also probably affects the peak at 3015 cm^−^^1^, which almost disappears (Figure 9a). A similar effect was recorded upon the interaction of dodecyl-ammonium and carrageenan [53]. Thus, the obtained FTIR spectra confirm the ionic interaction of CTAB and carrageenan.

The peak at 2847 cm^−^^1^ was used to estimate the cetyltrimethylammonium content in the formed ionic associates. The calibration line has fairly good linearity (Figure 9b). Figure 9c shows the content of cetyltrimethylammonium in the precipitate as determined with the above calibration. It can be seen that the composition of the precipitated ionic associates varies depending on the initial ratio of the reactants. This is consistent with the proposed mechanism of interaction between carrageenan and CTAB (Figure 6).

## 3. Materials and Methods

### 3.1. Reagents and Solutions

Cetyltrimethylammonium bromide (Koch-Light Laboratories, Haverhill, UK) and Kappa carrageenan (Sigma-Aldrich, Saint Louis, MO, USA) were used as received. The sulphur content in the carrageenan measured by ICP OES was 43.3 mg/g [24]. The 1 g/L aqueous carrageenan stock solution for sedimentation tests and 8 g/L aqueous carrageenan solution for titration tests were stored at 4 °C. The 20 mM solution of cetyltrimethylammonium bromide (CTAB) was prepared using aqueous methanol solution (36% *v*/*v*) as a solvent.

### 3.2. Suspension Sedimentation Tests

Sedimentation of the suspended particles was monitored with a Turbiscan LAB scanning turbidimeter (Formulaction, Toulouse, France). The instrument uses near-infrared light (880 nm) to ensure uniform registration of particles of various sizes and shapes. The suspensions were scanned vertically along the glass cell with a transmittance detector. The Turbiscan Stability Index (TSI) was calculated as follows:

$${TSI}_{t}=\left(\sum_{h=1}^{H}\left|{transmittance}_{0}(h)-{transmittance}_{t}(h)\right|\right)/H$$

where TSI_t_ is the index value at time t; transmittance_0_(h) is the initial transmittance at height h; transmittance_t_(h) is the transmittance at time t and at height h; H is the total number of heights under measurement.

### 3.3. Study of the Shape and Size of Suspended Particles

Suspensions were prepared by mixing CTAB and carrageenan stock solutions in the specified proportions. Polarised light micro-images were made using a B-500 optical microscope (Optika, Ponteranica, Italy). Particle size distributions were determined using a laser particle sizer ANALYSETTE 22 (Fritsch, Idar-Oberstein, Germany).

### 3.4. Turbidimetric Titrations

Titrations were carried out in a 200 mL beaker with constant stirring, adding 5 to 50 μL aliquots at 10 s intervals using a Titronic 500 piston burette (SI Analytics, Mainz, Germany). An exact volume of 100 mL of CTAB solution (range: 0.6 to 2 mM) was titrated with 8 g/L carrageenan solution. Alternatively, exactly 100 mL of the carrageenan solution (range: 0.007 to 2.100 g/L) was titrated with 20 mM CTAB solution. Turbidity changes were measured with an IR photodetector set at 90° to the IR beam from a 1 W LED emitting at 850 nm. Burette control and signal acquisition were performed using custom ChemiON software (J. Lamkiewicz [32]).

### 3.5. Study of Structure and Composition of Ion Pair Associates

The stock solutions of carrageenan and CTAB were mixed at the specified proportions, the resulting suspensions were centrifuged and the precipitates were dried at 60 °C. X-ray diffractograms were recorded using a Seifert diffractometer (Aldersbach, Germany) with a CuKalpha source and a nickel filter. ATR-FTIR spectra were recorded using an Alpha-P spectrometer (Bruker, Billerica, MA, USA) with the diamond window.

## 4. Conclusions

The spontaneous flocculation of ionic associates with different proportions of carrageenan and cetyltrimethylammonium was investigated. When the amounts of the reactants were far from equal, the excess component provided additional charge, and the ionic associates being formed were stable colloids. When the amounts of carrageenan mers and cetyltrimethylammonium ions were almost equimolar, the colloidal particles lost their charge and auto-flocculated. The proposed probable mechanism takes into account both ionic and hydrophobic interactions. The resulting flocs did not have well-defined edges, so polarised light micro-images were used to estimate their shape and size. The irregular shapes of the flocs confirm that they were aggregates of lesser particles.

The effect of component ratio on flocculation was investigated by recording sedimentation profiles and particle size distributions. The resulting suspensions were found to be fully stable when the ratio of cetyltrimethylammonium to carrageenan was ≤1 or ≥5 mmol/g. The most intensive flocculation occurred when the ratio was in the range of 1.7 to 2 mmol/g. The critical ratio values were also precisely determined using the turbidimetric titration technique. The turbidity plots contain marked break points, indicating the most extensive flocculation. The relationships between the amounts of cetyltrimethylammonium and carrageenan at the break point are strictly linear plots (R^2^ ≥ 0.999), indicating that the critical ratio is a constant value. The critical ratio was found at 1.53 and 1.78 mmol/g, respectively, during the titration of carrageenan with CTAB and vice versa. These values are rather consistent with the number of anionic sulfate groups in carrageenan (1.35 mmol/g) as measured by ICP OES. The value of 1.78 mmol/g is within the range of 1.7 to 2 mmol/g obtained from Turbiscan measurements, while the value of 1.53 mmol/g is outside the range. The two different critical component ratios clearly indicate that the order in which components are added plays a role in the formation of flocs.

The results may be used to develop a method for removing toxic CTAB residues from solutions. Introducing carrageenan into the CTAB solution while maintaining appropriate quantitative proportions will cause rapid precipitation of the associate, which can then be separated from the mixture using filtration methods.

## Data Availability

All data is contained within the article.
